# Ten game-changers in mental health for South Africa

**DOI:** 10.4102/sajpsychiatry.v29i0.2180

**Published:** 2023-11-06

**Authors:** Dan J. Stein, Gustaaf G. Wolvaardt, Nompumelelo Zungu, Olive Shisana

**Affiliations:** 1 Department of Psychiatry and Mental Health, Faculty of Health Sciences, University of Cape Town, Cape Town, South Africa; 2 Neuroscience Institute, Faculty of Health Sciences, University of Cape Town, Cape Town, South Africa; 3 MRC Unit on Risk and Resilience in Mental Disorders, Faculty of Health Sciences, University of Cape Town, Cape Town, South Africa; 4 Foundation for Professional Development, Tshwane, South Africa; 5 Public Health, Society and Belonging Division, Human Sciences Research Council of South Africa, Pretoria, South Africa; 6 School of Nursing and Public Health, University of KwaZulu-Natal, Durban, South Africa

The dawning of democracy in South Africa provided the opportunity for a broad range of new policies, including in health and mental health. The *Mental Health Act of 2002* embodied much needed transformation in the sector. The Ekurhuleni Declaration of 2012 articulated a comprehensive and compelling vision for mental health. Nevertheless, shortly thereafter the Life Esidimeni tragedy occurred, with the death of more than 140 individuals with mental illness, exposing a range of quality deficits.^1^ This demonstrated that there is a disjuncture between the reality on the ground and the ideals of mental health policies and visions. Thus, renewed attention to strategy and implementation is needed.

To this end, a South African Mental Health conference was initiated by the Foundation for Professional Development (FPD), working with the Department of Health and the scientific community. The conference built on the experience of FPD in conferences addressing human immunodeficiency virus (HIV) and/or acquired immune deficiency syndrome (AIDS) and tuberculosis (TB). The conference coincided with the release of the Department of Health’s National Mental Health Policy Framework (2023–2030) and had a broad range of participants, including members of national and provincial Departments of Health, non-governmental organisations (NGOs), clinicians, researchers, persons with lived experience of mental health disorders and patient advocates. In this editorial, drawing on work presented at the conference, we outline 10 game-changers for mental health in the country.

Two game-changers are consistent with an emphasis of a whole-of-society approach. Firstly, we need to further consolidate the move from the World Health Organization’s 4 × 4 framework (emphasising four conditions [cardiovascular, cancer, chronic respiratory disease, diabetes] and four risk factors [diet, inactivity, alcohol, tobacco]) to a 5 × 5 framework that includes mental health as an additional condition and early trauma as a 5th risk factor.^2^ While there are multiple risk factors for mental disorders, key interventions under this framework might include harm reduction for substance use and a focus on the first 1000 days.^3^ Secondly, we need to recognise that provision of such basics as electricity and safety, as well as steps to alleviate poverty, are crucial for mental health.^4^ Conversely, mental health is crucial for sustainable development.^5^

A second set of game-changers relates to authority given to governance institutions. Firstly, we need to provide more independence and more bite to monitoring bodies that have been set up by our legislature. The Central Drug Authority, for example, plays a key role in developing a National Drug Master Plan for the country, but it falls under the umbrella of a single department (Social Development) and has a small budget, so drastically constraining implementation. Similarly, Mental Health Review Boards fall under the umbrella of the *Mental Health Care Act* but have little ability to insist on better resources for patients. Secondly, a range of institutions, including the universities, the Human Sciences Research Council, the South African Medical Research Council and the National Foundation for Research need to adopt specific goals for increasing training and research in mental health.

A third set of game-changers involves human resourcing. Limitations in the mental health workforce contribute to a significant treatment gap.^6^ A growing body of literature has emphasised that many mental health interventions can be provided by counsellors and non-specialised health workers.^7,8^ We need to move away from a focus on guarding professional turf and towards a focus on competency-based care provided by a range of practitioners.^9^ Examples of this include the creation of many more counsellor posts in clinics, schools and communities. Furthermore, we need parity of human resourcing: if district hospitals can afford beds run by obstetricians and paediatricians as well as nurses focused on these specialities, then they must employ at least equal numbers of psychiatrists, clinical psychologists and allied mental health professionals. Only a minority of patients with mental disorders should be referred to centralised psychiatric hospitals.

A fourth set of game-changers entails sharpening our focus. Mental health services need to determine key indicators and to address them robustly. The response to the HIV epidemic has benefited enormously by setting clear targets and timelines, such as the 95:95:95 indicators.^10^ We need to follow our colleagues, choosing indicators that are feasible to monitor and acceptable to those with lived experience and then ensure their promotion and targeting. In addition, we need to better address overlooked and vulnerable populations, including those with intellectual disability, neuro-HIV and/or neuro-AIDS, substance use, children and adolescents, the elderly and pregnant women. In line with earlier comments about human resourcing, it is remarkable that tertiary hospitals in South Africa employ Health Professions Council of South Africa (HPCSA) registered sub-speciality physicians and surgeons, but vanishingly few are able to care for key vulnerable populations using HPCSA-registered addictionologists, neuropsychiatrists and geriatric mental health specialists.^11^

A final set of game-changers pertains to the health and mental health professions. As above, we need to move away from guarding professional turf and towards competency-based care.^9^ Nurse prescription of psychotropic agents is surely needed in order to ensure access to effective interventions for mental disorders throughout the country. Most individuals with mental disorders are seen by non-mental health specialists in general health settings. We therefore need to ensure that all health professionals, and particularly mental health professionals, are delivering evidence-based care effectively, including interventions such as psychoeducation, motivational interviewing and problem-solving therapy.^12^ Collaborative care in particular deserves to be widely rolled out.^13^

Arguably, all 10 of these game-changers can be collapsed into one key idea. This is funding and implementation by Provinces of the National Mental Health Policy Framework 2023–2030. As sadly demonstrated by Life Esidimeni, excellent policy frameworks require appropriate funding and rigorous implementation.^14^ Unfortunately, there is little evidence that the National Mental Health Policy Framework of 2013–2020 led to such funding and implementation or improved mental health outcomes. On the contrary, we must fully acknowledge and clearly address examples of death by maladministration.^15^

Furthermore, there is nothing magical about the number ‘10’. Here we have focused largely on issues related to implementation of national policy. However, there are multiple factors that contribute to mental disorders, and there are therefore a wide range of potential interventions that ought to be put on the table. Interventions such as book-sharing for children, resilience building for adolescents, peer-delivered counselling for students and the friendship bench at institutions deserve consideration.^16,17^ A range of institutions, including universities, ought to adopt mental health strategies, and develop learning-based programmes to improve mental health. Strengthening the work done by NGOs working in the mental health space, by psychiatric hospitals and by provincial mental health co-ordinators may well be useful. There is significant scope for employing digital methodologies.^18^

In summary, if mental health services are to improve in South Africa, good national mental health policies need to be supplemented by ongoing mental health advocacy. Key issues are appropriate budgeting for and rigorous implementation of the national Mental Health Policy Framework. Such budgeting and implementation would allow for better mental health services. Some matters, such as improved electricity and safety, are whole-of-society issues where mental health clinicians have no particular expertise. With regard to some issues, such as strengthening institutional responses and improving human resourcing, we have expertise but no particular clout. And for yet other matters, including improving the employment of evidence-based interventions, encouraging competency-based health systems and conducting appropriate research, mental health clinicians can be major leaders and drivers.
